# Hypoallergenic allergen derivatives of Pru p 3 for immunotherapy of IgE-mediated peach allergy

**DOI:** 10.1186/2045-7022-3-S3-P177

**Published:** 2013-07-25

**Authors:** B Linhart, A Gstoettner, C Gamez, I Swoboda, A Mari, N Papadopoulos, R Valenta

**Affiliations:** 1 Dept. of Pathophysiology and Allergy Research, Medical University of Vienna, Vienna, Austria; 2 Institute for Medical Research, Foundation Jimenez Diaz, Madrid, Spain; 3 Center for Molecular Allergology, IDI-IRCCS, Rome, Italy; 4 Allergy Research Center, 2nd Pediatric Department, National & Kapodistrian University of Athens, Athens, Greece

## Background

IgE-mediated allergy to peach represents a frequent cause of severe anaphylactic reactions in Mediterranean countries. Pru p 3, a nonspecific lipid transfer protein (LTP) from peach, was identified as an important elicitor of IgE-mediated food hypersensitivity reactions to peach. Specific immunotherapy is currently the only allergen-specific and disease-modifying treatment of allergies, but has so far not been applied to food allergic patients due to the high risk of IgE-mediated side effects. Hypoallergenic Pru p 3 derivatives have therefore been constructed in order to minimize side-effects, but these molecules were not immunogenic in animal models.

## Methods

Two recombinant Pru p 3-derivatives were developed based on the Pru p 3 amino acid sequence, by introduction of point mutations, or rearrangements of Pru p 3 fragments and oligomerization. Genes encoding the resulting Pru p 3 derivatives were cloned into the expression vector pET27b and expressed in *Escherichia coli*. The proteins were purified by affinity chromatography and analyzed by SDS-PAGE and mass spectrometry. IgE-reactivity was tested by dot blot analysis and immunogenicity was investigated by immunization of rabbits.

## Results

Expression of the two recombinant Pru p 3 derivatives in *Escherichia coli* yielded high amounts of recombinant proteins. They were purified to homogeneity and exhibited a strongly reduced IgE-reactivity when exposed to sera of peach allergic patients. Upon immunization of rabbits the Pru p 3 derivatives induced a robust IgG antibody response specific for natural Pru p 3.

## Conclusion

The reported recombinant hypoallergenic Pru p 3 derivatives are candidates for specific immunotherapy of peach allergic patients.

## Disclosure of interest

B Linhart: None declared, A Gstoettner: None declared, C Gamez: None declared, I Swoboda: None declared, A Mari: None declared, N Papadopoulos: None declared, R Valenta: Grant/research support from Biomay AG, Vienna, Austria.

